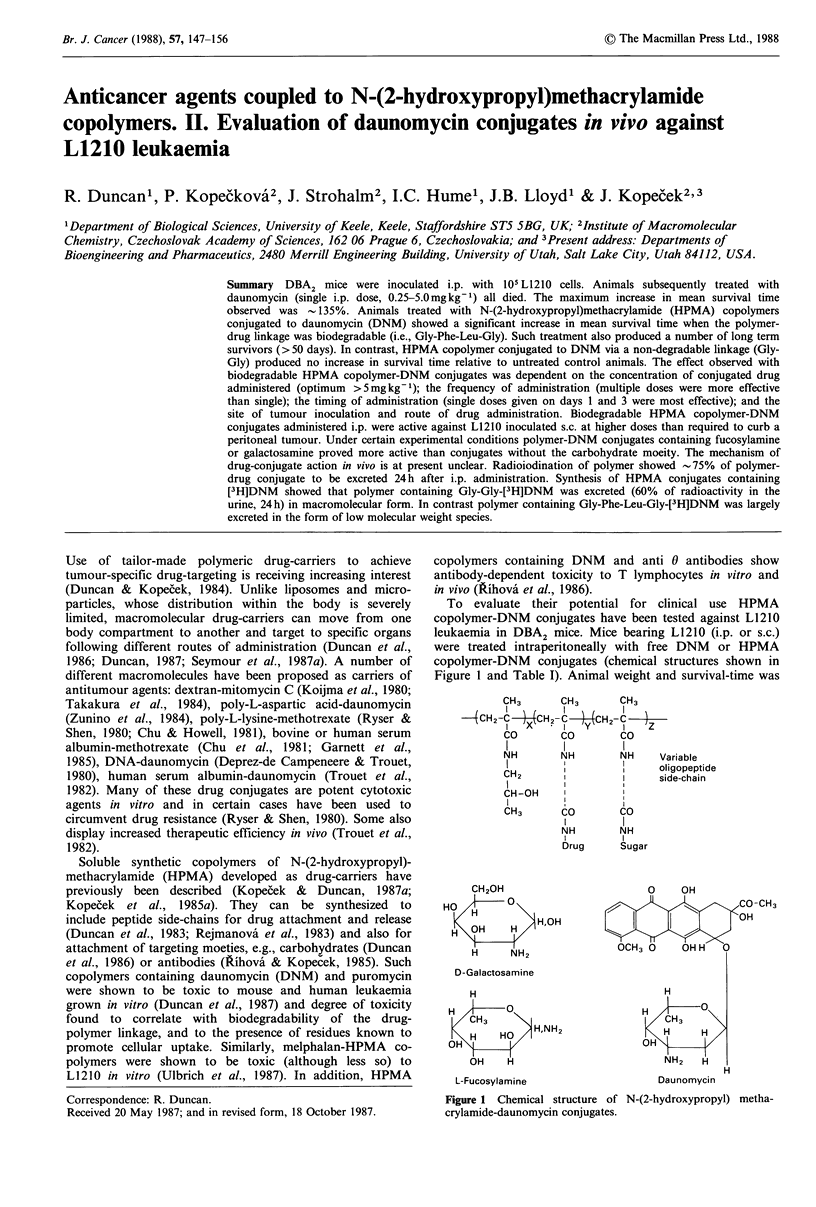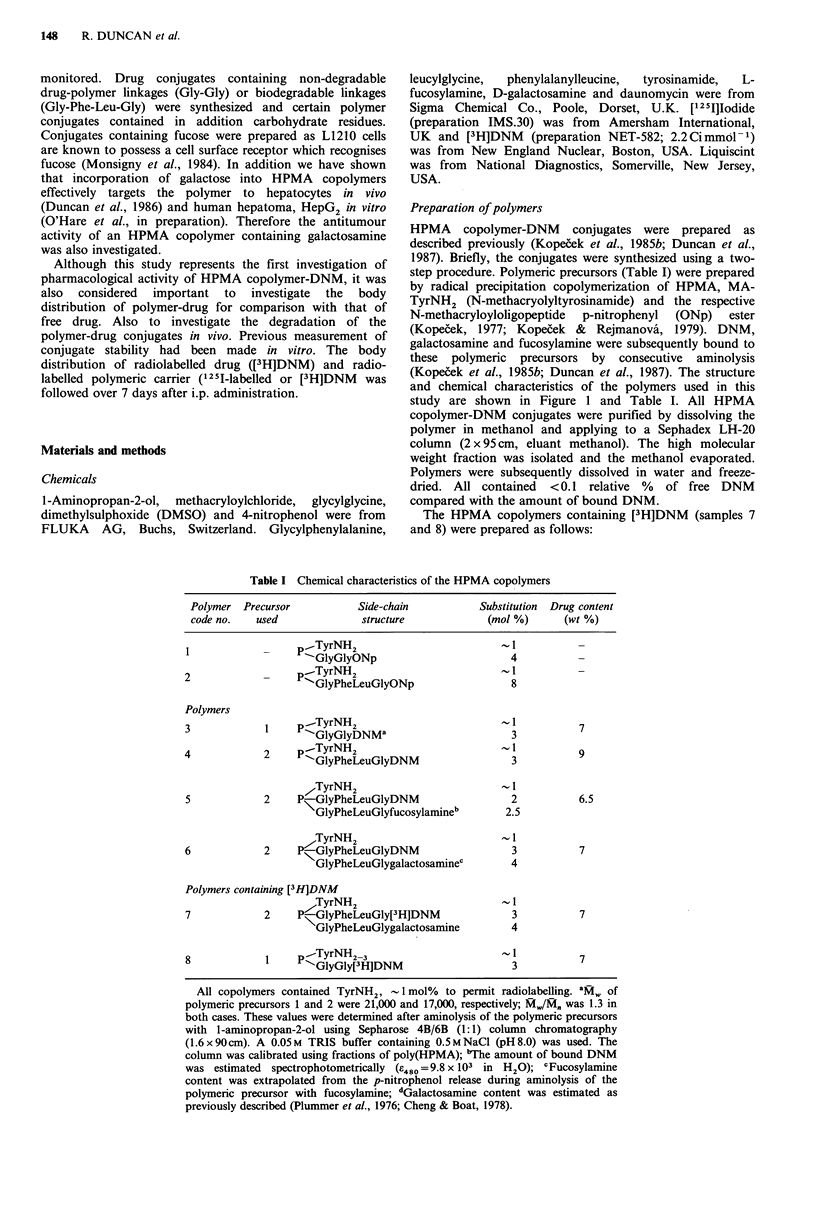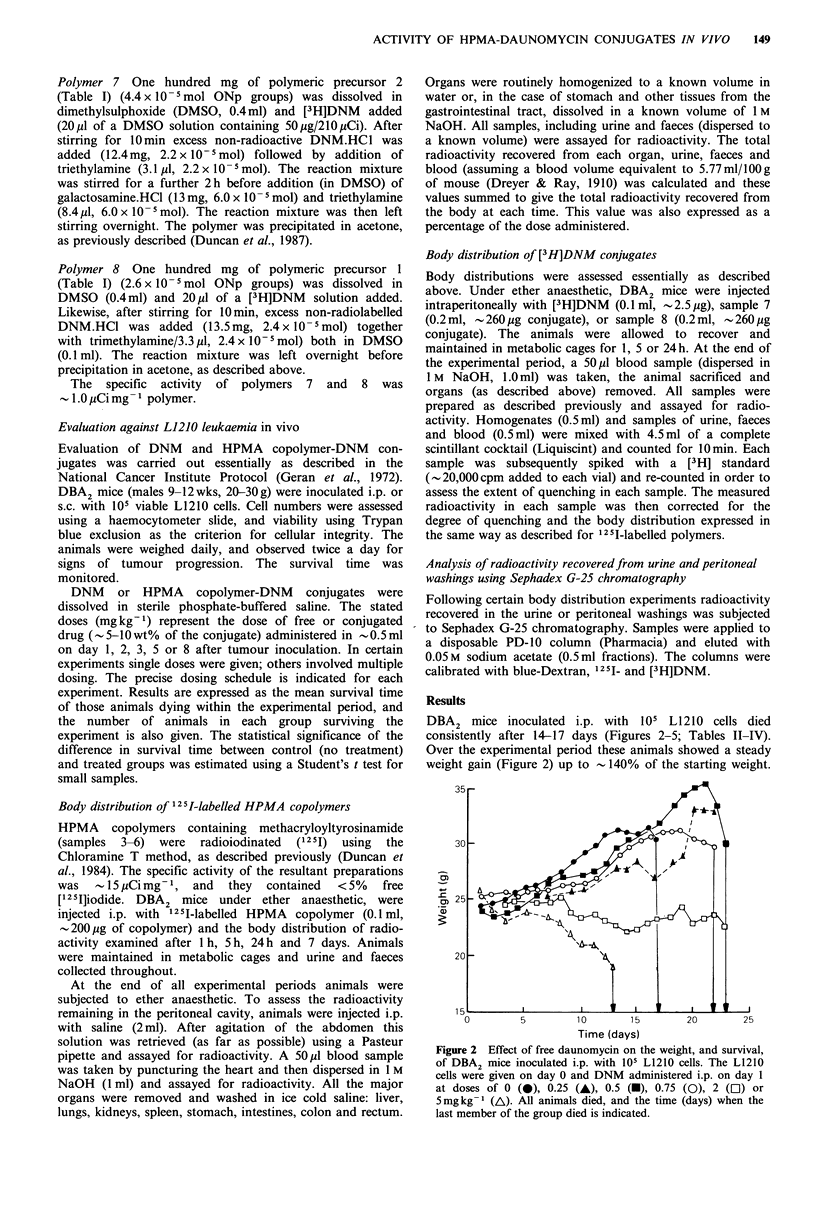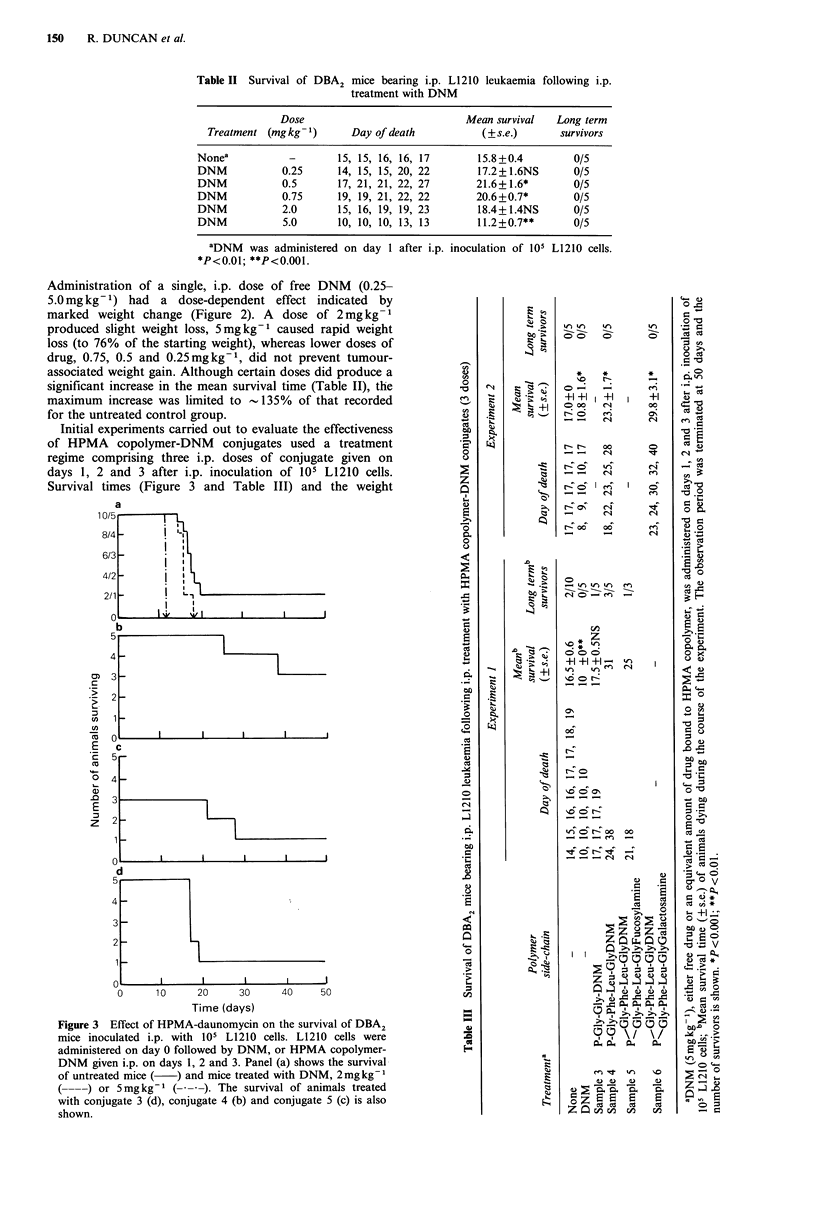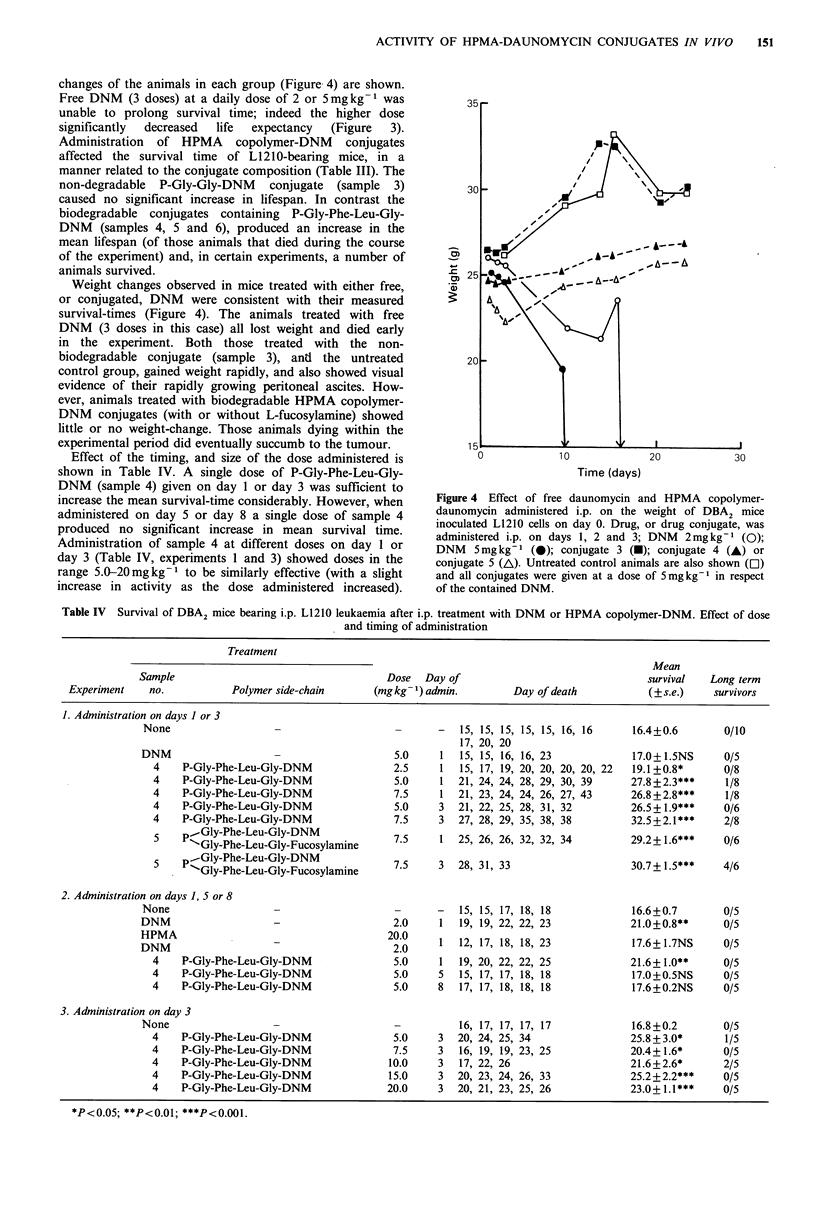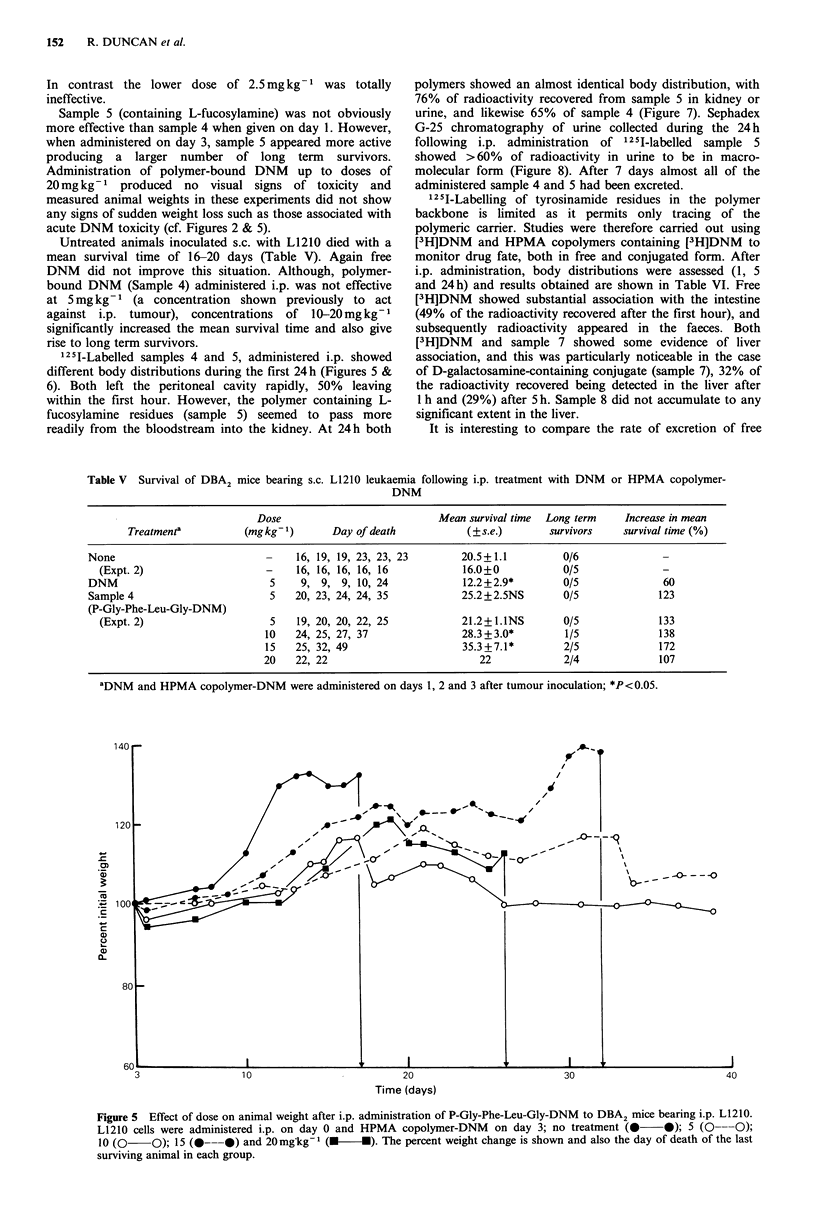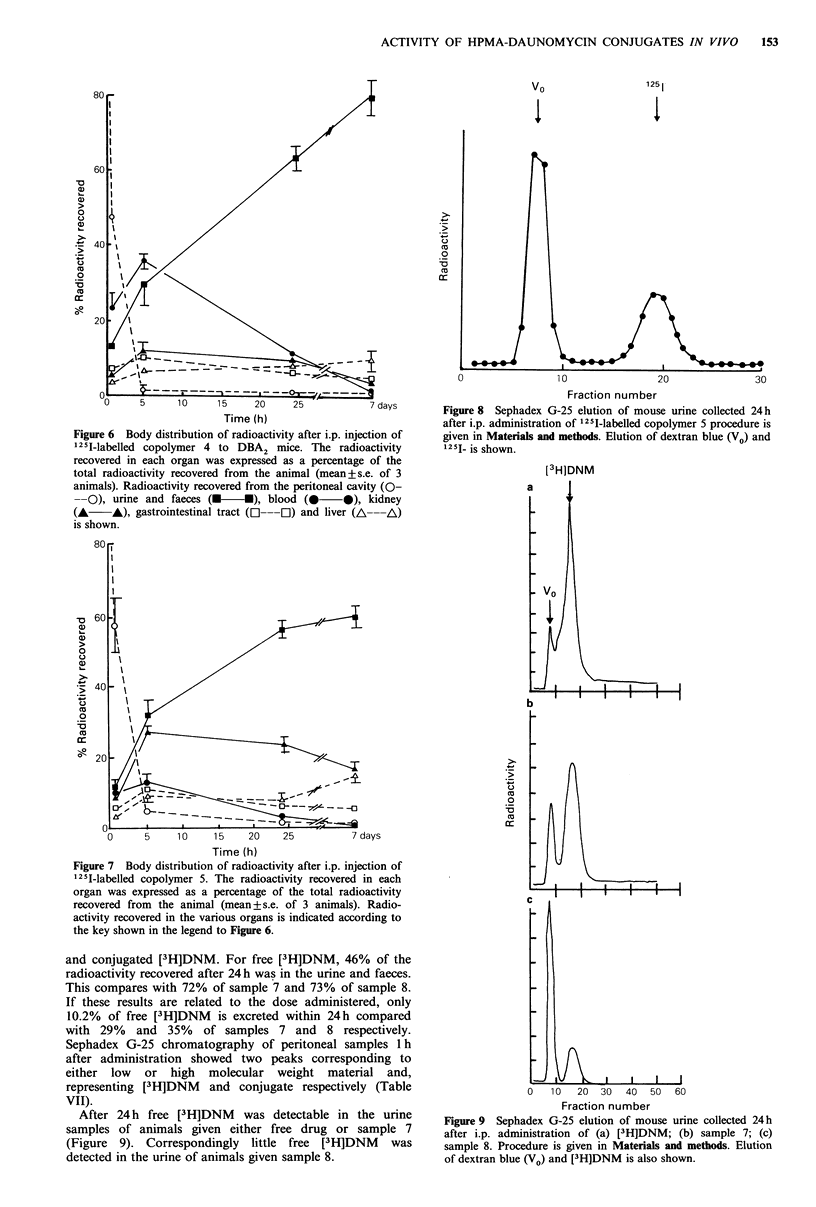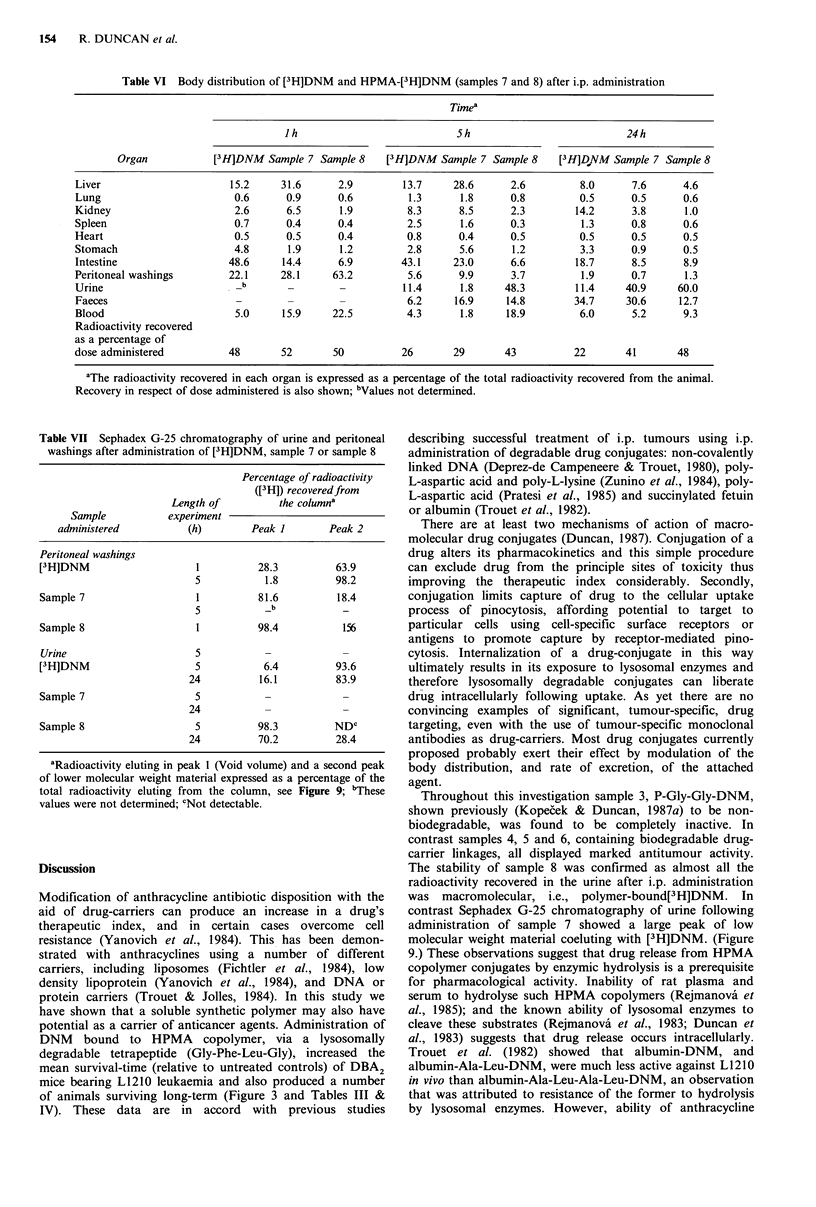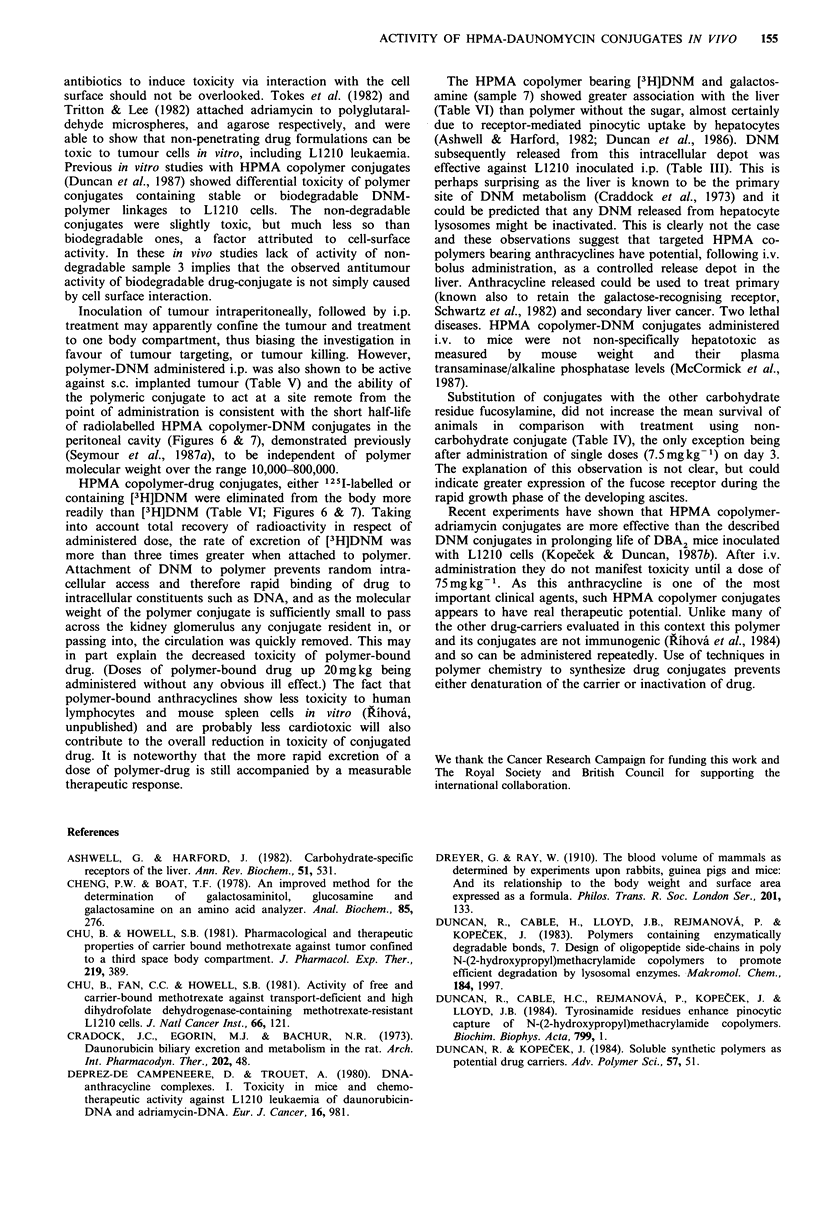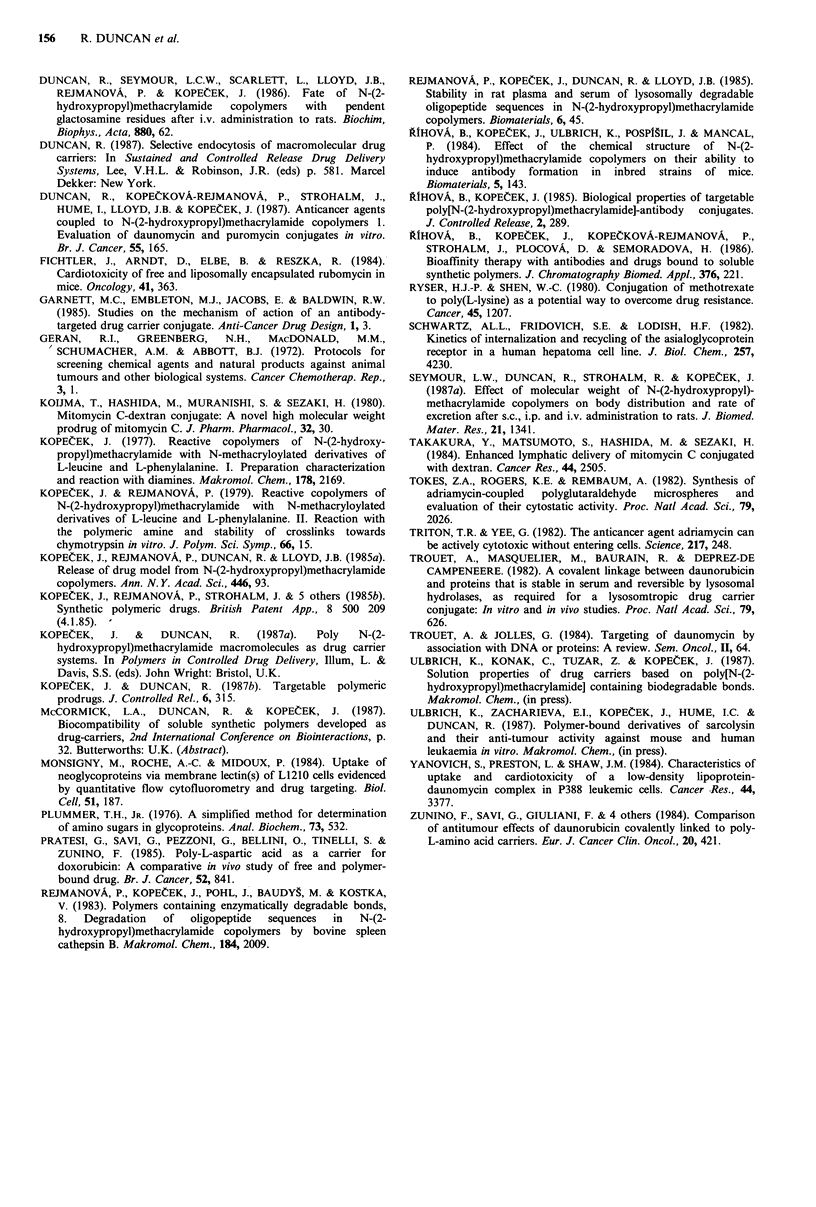# Anticancer agents coupled to N-(2-hydroxypropyl)methacrylamide copolymers. II. Evaluation of daunomycin conjugates in vivo against L1210 leukaemia.

**DOI:** 10.1038/bjc.1988.31

**Published:** 1988-02

**Authors:** R. Duncan, P. KopeckovÃ¡, J. Strohalm, I. C. Hume, J. B. Lloyd, J. Kopecek

**Affiliations:** Department of Biological Sciences, University of Keele, Staffordshire, UK.

## Abstract

DBA2 mice were inoculated i.p. with 10(5)L1210 cells. Animals subsequently treated with daunomycin (single i.p. dose, 0.25-5.0 mg kg-1) all died. The maximum increase in mean survival time observed was approximately 135%. Animals treated with N-(2-hydroxypropyl)methacrylamide (HPMA) copolymers conjugated to daunomycin (DNM) showed a significant increase in mean survival time when the polymer-drug linkage was biodegradable (i.e., Gly-Phe-Leu-Gly). Such treatment also produced a number of long term survivors (greater than 50 days). In contrast, HPMA copolymer conjugated to DNM via a non-degradable linkage (Gly-Gly) produced no increase in survival time relative to untreated control animals. The effect observed with biodegradable HPMA copolymer-DNM conjugates was dependent on the concentration of conjugated drug administered (optimum greater than 5 mg kg-1); the frequency of administration (multiple doses were more effective than single); the timing of administration (single doses given on days 1 and 3 were most effective); and the site of tumour inoculation and route of drug administration. Biodegradable HPMA copolymer-DNM conjugates administered i.p. were active against L1210 inoculated s.c. at higher doses than required to curb a peritoneal tumour. Under certain experimental conditions polymer-DNM conjugates containing fucosylamine or galactosamine proved more active than conjugates without the carbohydrate moeity. The mechanism of drug-conjugate action in vivo is at present unclear. Radioiodination of polymer showed approximately 75% of polymer-drug conjugate to be excreted 24 h after i.p. administration. Synthesis of HPMA conjugates containing [3H]DNM showed that polymer containing Gly-Gly-[3H]DNM was excreted (60% of radioactivity in the urine, 24 h) in macromolecular form. In contrast polymer containing Gly-Phe-Leu-Gly-[3H]DNM was largely excreted in the form of low molecular weight species.


Br. J. Cancer (1988), 57, 147–156 © The Macmillan Press Ltd., 1988

### Anticancer agents coupled to N-(2-hydroxypropyl)methacrylamide copolymers. II. Evaluation of daunomycin conjugates *in vivo* against L1210 leukaemia

R. Duncan[1], P. Kopečková[2], J. Strohalm[2], I.C. Hume[1], J.B. Lloyd[1] & J. Kopeček[2,3]

[1]*Department of Biological Sciences, University of Keele, Keele, Staffordshire ST5 5BG, UK;* [2]*Institute of Macromolecular Chemistry, Czechoslovak Academy of Sciences, 162 06 Prague 6, Czechoslovakia; and* [3]*Present address: Departments of Bioengineering and Pharmaceutics, 2480 Merrill Engineering Building, University of Utah, Salt Lake City, Utah 84112, USA.*

**Summary** $DBA_2$ mice were inoculated i.p. with $10^5$ L1210 cells. Animals subsequently treated with daunomycin (single i.p. dose, 0.25–5.0 mg kg$^{-1}$) all died. The maximum increase in mean survival time observed was ~135%. Animals treated with N-(2-hydroxypropyl)methacrylamide (HPMA) copolymers conjugated to daunomycin (DNM) showed a significant increase in mean survival time when the polymer-drug linkage was biodegradable (i.e., Gly-Phe-Leu-Gly). Such treatment also produced a number of long term survivors (>50 days). In contrast, HPMA copolymer conjugated to DNM via a non-degradable linkage (Gly-Gly) produced no increase in survival time relative to untreated control animals. The effect observed with biodegradable HPMA copolymer-DNM conjugates was dependent on the concentration of conjugated drug administered (optimum >5 mg kg$^{-1}$); the frequency of administration (multiple doses were more effective than single); the timing of administration (single doses given on days 1 and 3 were most effective); and the site of tumour inoculation and route of drug administration. Biodegradable HPMA copolymer-DNM conjugates administered i.p. were active against L1210 inoculated s.c. at higher doses than required to curb a peritoneal tumour. Under certain experimental conditions polymer-DNM conjugates containing fucosylamine or galactosamine proved more active than conjugates without the carbohydrate moeity. The mechanism of drug-conjugate action *in vivo* is at present unclear. Radioiodination of polymer showed ~75% of polymer-drug conjugate to be excreted 24 h after i.p. administration. Synthesis of HPMA conjugates containing [$^3$H]DNM showed that polymer containing Gly-Gly-[$^3$H]DNM was excreted (60% of radioactivity in the urine, 24 h) in macromolecular form. In contrast polymer containing Gly-Phe-Leu-Gly-[$^3$H]DNM was largely excreted in the form of low molecular weight species.

Use of tailor-made polymeric drug-carriers to achieve tumour-specific drug-targeting is receiving increasing interest (Duncan & Kopeček, 1984). Unlike liposomes and micro-particles, whose distribution within the body is severely limited, macromolecular drug-carriers can move from one body compartment to another and target to specific organs following different routes of administration (Duncan *et al.*, 1986; Duncan, 1987; Seymour *et al.*, 1987*a*). A number of different macromolecules have been proposed as carriers of antitumour agents: dextran-mitomycin C (Koijma *et al.*, 1980; Takakura *et al.*, 1984), poly-L-aspartic acid-daunomycin (Zunino *et al.*, 1984), poly-L-lysine-methotrexate (Ryser & Shen, 1980; Chu & Howell, 1981), bovine or human serum albumin-methotrexate (Chu *et al.*, 1981; Garnett *et al.*, 1985), DNA-daunomycin (Deprez-de Campeneere & Trouet, 1980), human serum albumin-daunomycin (Trouet *et al.*, 1982). Many of these drug conjugates are potent cytotoxic agents *in vitro* and in certain cases have been used to circumvent drug resistance (Ryser & Shen, 1980). Some also display increased therapeutic efficiency *in vivo* (Trouet *et al.*, 1982).

Soluble synthetic copolymers of N-(2-hydroxypropyl)-methacrylamide (HPMA) developed as drug-carriers have previously been described (Kopeček & Duncan, 1987*a*; Kopeček *et al.*, 1985*a*). They can be synthesized to include peptide side-chains for drug attachment and release (Duncan *et al.*, 1983; Rejmanová *et al.*, 1983) and also for attachment of targeting moeties, e.g., carbohydrates (Duncan *et al.*, 1986) or antibodies (Říhová & Kopeček, 1985). Such copolymers containing daunomycin (DNM) and puromycin were shown to be toxic to mouse and human leukaemia grown *in vitro* (Duncan *et al.*, 1987) and degree of toxicity found to correlate with biodegradability of the drug-polymer linkage, and to the presence of residues known to promote cellular uptake. Similarly, melphalan-HPMA co-polymers were shown to be toxic (although less so) to L1210 *in vitro* (Ulbrich *et al.*, 1987). In addition, HPMA copolymers containing DNM and anti θ antibodies show antibody-dependent toxicity to T lymphocytes *in vitro* and *in vivo* (Říhová *et al.*, 1986).

To evaluate their potential for clinical use HPMA copolymer-DNM conjugates have been tested against L1210 leukaemia in $DBA_2$ mice. Mice bearing L1210 (i.p. or s.c.) were treated intraperitoneally with free DNM or HPMA copolymer-DNM conjugates (chemical structures shown in Figure 1 and Table I). Animal weight and survival-time was

**Figure 1** Chemical structure of N-(2-hydroxypropyl) methacrylamide-daunomycin conjugates.

Correspondence: R. Duncan.
Received 20 May 1987; and in revised form, 18 October 1987.

monitored. Drug conjugates containing non-degradable drug-polymer linkages (Gly-Gly) or biodegradable linkages (Gly-Phe-Leu-Gly) were synthesized and certain polymer conjugates contained in addition carbohydrate residues. Conjugates containing fucose were prepared as L1210 cells are known to possess a cell surface receptor which recognises fucose (Monsigny *et al.*, 1984). In addition we have shown that incorporation of galactose into HPMA copolymers effectively targets the polymer to hepatocytes *in vivo* (Duncan *et al.*, 1986) and human hepatoma, HepG$_2$ *in vitro* (O'Hare *et al.*, in preparation). Therefore the antitumour activity of an HPMA copolymer containing galactosamine was also investigated.

Although this study represents the first investigation of pharmacological activity of HPMA copolymer-DNM, it was also considered important to investigate the body distribution of polymer-drug for comparison with that of free drug. Also to investigate the degradation of the polymer-drug conjugates *in vivo*. Previous measurement of conjugate stability had been made *in vitro*. The body distribution of radiolabelled drug ([$^3$H]DNM) and radiolabelled polymeric carrier ($^{125}$I-labelled or [$^3$H]DNM was followed over 7 days after i.p. administration.

### Materials and methods

### *Chemicals*

1-Aminopropan-2-ol, methacryloylchloride, glycylglycine, dimethylsulphoxide (DMSO) and 4-nitrophenol were from FLUKA AG, Buchs, Switzerland. Glycylphenylalanine, leucylglycine, phenylalanylleucine, tyrosinamide, L-fucosylamine, D-galactosamine and daunomycin were from Sigma Chemical Co., Poole, Dorset, U.K. [$^{125}$I]Iodide (preparation IMS.30) was from Amersham International, UK and [$^3$H]DNM (preparation NET-582; 2.2 Ci mmol$^{-1}$) was from New England Nuclear, Boston, USA. Liquiscint was from National Diagnostics, Somerville, New Jersey, USA.

### *Preparation of polymers*

HPMA copolymer-DNM conjugates were prepared as described previously (Kopeček *et al.*, 1985*b*; Duncan *et al.*, 1987). Briefly, the conjugates were synthesized using a two-step procedure. Polymeric precursors (Table I) were prepared by radical precipitation copolymerization of HPMA, MA-TyrNH$_2$ (N-methacryolyltyrosinamide) and the respective N-methacryloyloligopeptide p-nitrophenyl (ONp) ester (Kopeček, 1977; Kopeček & Rejmanová, 1979). DNM, galactosamine and fucosylamine were subsequently bound to these polymeric precursors by consecutive aminolysis (Kopeček *et al.*, 1985*b*; Duncan *et al.*, 1987). The structure and chemical characteristics of the polymers used in this study are shown in Figure 1 and Table I. All HPMA copolymer-DNM conjugates were purified by dissolving the polymer in methanol and applying to a Sephadex LH-20 column (2 x 95 cm, eluant methanol). The high molecular weight fraction was isolated and the methanol evaporated. Polymers were subsequently dissolved in water and freeze-dried. All contained <0.1 relative % of free DNM compared with the amount of bound DNM.

The HPMA copolymers containing [$^3$H]DNM (samples 7 and 8) were prepared as follows:

**Table I** Chemical characteristics of the HPMA copolymers

| *Polymer code no.* | *Precursor used* | *Side-chain structure* | *Substitution (mol %)* | *Drug content (wt %)* |
|---|---|---|---|---|
| 1 | – | P<TyrNH$_2$<br>GlyGlyONp | ~1<br>4 | –<br>– |
| 2 | – | P<TyrNH$_2$<br>GlyPheLeuGlyONp | ~1<br>8 | – |
| *Polymers* | | | | |
| 3 | 1 | P<TyrNH$_2$<br>GlyGlyDNM[a] | ~1<br>3 | 7 |
| 4 | 2 | P<TyrNH$_2$<br>GlyPheLeuGlyDNM | ~1<br>3 | 9 |
| 5 | 2 | P<TyrNH$_2$<br>GlyPheLeuGlyDNM<br>GlyPheLeuGlyfucosylamine[b] | ~1<br>2<br>2.5 | 6.5 |
| 6 | 2 | P<TyrNH$_2$<br>GlyPheLeuGlyDNM<br>GlyPheLeuGlygalactosamine[c] | ~1<br>3<br>4 | 7 |
| *Polymers containing [$^3$H]DNM* | | | | |
| 7 | 2 | P<TyrNH$_2$<br>GlyPheLeuGly[$^3$H]DNM<br>GlyPheLeuGlygalactosamine | ~1<br>3<br>4 | 7 |
| 8 | 1 | P<TyrNH$_{2-3}$<br>GlyGly[$^3$H]DNM | ~1<br>3 | 7 |

All copolymers contained TyrNH$_2$, ~1 mol% to permit radiolabelling. [a]$\bar{M}_w$ of polymeric precursors 1 and 2 were 21,000 and 17,000, respectively; $\bar{M}_w/\bar{M}_n$ was 1.3 in both cases. These values were determined after aminolysis of the polymeric precursors with 1-aminopropan-2-ol using Sepharose 4B/6B (1:1) column chromatography (1.6 x 90 cm). A 0.05 M TRIS buffer containing 0.5 M NaCl (pH 8.0) was used. The column was calibrated using fractions of poly(HPMA); [b]The amount of bound DNM was estimated spectrophotometrically ($\varepsilon_{480} = 9.8 \times 10^3$ in $H_2O$); [c]Fucosylamine content was extrapolated from the *p*-nitrophenol release during aminolysis of the polymeric precursor with fucosylamine; [d]Galactosamine content was estimated as previously described (Plummer *et al.*, 1976; Cheng & Boat, 1978).

*Polymer 7* One hundred mg of polymeric precursor 2 (Table I) ($4.4 \times 10^{-5}$ mol ONp groups) was dissolved in dimethylsulphoxide (DMSO, 0.4 ml) and [$^3$H]DNM added (20 $\mu$l of a DMSO solution containing 50 $\mu$g/210 $\mu$Ci). After stirring for 10 min excess non-radioactive DNM.HCl was added (12.4 mg, $2.2 \times 10^{-5}$ mol) followed by addition of triethylamine (3.1 $\mu$l, $2.2 \times 10^{-5}$ mol). The reaction mixture was stirred for a further 2 h before addition (in DMSO) of galactosamine.HCl (13 mg, $6.0 \times 10^{-5}$ mol) and triethylamine (8.4 $\mu$l, $6.0 \times 10^{-5}$ mol). The reaction mixture was then left stirring overnight. The polymer was precipitated in acetone, as previously described (Duncan *et al.*, 1987).

*Polymer 8* One hundred mg of polymeric precursor 1 (Table I) ($2.6 \times 10^{-5}$ mol ONp groups) was dissolved in DMSO (0.4 ml) and 20 $\mu$l of a [$^3$H]DNM solution added. Likewise, after stirring for 10 min, excess non-radiolabelled DNM.HCl was added (13.5 mg, $2.4 \times 10^{-5}$ mol) together with trimethylamine/3.3 $\mu$l, $2.4 \times 10^{-5}$ mol) both in DMSO (0.1 ml). The reaction mixture was left overnight before precipitation in acetone, as described above.

The specific activity of polymers 7 and 8 was $\sim 1.0\ \mu$Ci mg$^{-1}$ polymer.

*Evaluation against L1210 leukaemia* in vivo

Evaluation of DNM and HPMA copolymer-DNM conjugates was carried out essentially as described in the National Cancer Institute Protocol (Geran *et al.*, 1972). $DBA_2$ mice (males 9–12 wks, 20–30 g) were inoculated i.p. or s.c. with $10^5$ viable L1210 cells. Cell numbers were assessed using a haemocytometer slide, and viability using Trypan blue exclusion as the criterion for cellular integrity. The animals were weighed daily, and observed twice a day for signs of tumour progression. The survival time was monitored.

DNM or HPMA copolymer-DNM conjugates were dissolved in sterile phosphate-buffered saline. The stated doses (mg kg$^{-1}$) represent the dose of free or conjugated drug ($\sim$5–10 wt% of the conjugate) administered in $\sim$0.5 ml on day 1, 2, 3, 5 or 8 after tumour inoculation. In certain experiments single doses were given; others involved multiple dosing. The precise dosing schedule is indicated for each experiment. Results are expressed as the mean survival time of those animals dying within the experimental period, and the number of animals in each group surviving the experiment is also given. The statistical significance of the difference in survival time between control (no treatment) and treated groups was estimated using a Student's *t* test for small samples.

*Body distribution of* $^{125}$*I-labelled HPMA copolymers*

HPMA copolymers containing methacryloyltyrosinamide (samples 3–6) were radioiodinated ($^{125}$I) using the Chloramine T method, as described previously (Duncan *et al.*, 1984). The specific activity of the resultant preparations was $\sim 15\ \mu$Ci mg$^{-1}$, and they contained $<5\%$ free [$^{125}$I]iodide. $DBA_2$ mice under ether anaesthetic, were injected i.p. with $^{125}$I-labelled HPMA copolymer (0.1 ml, $\sim$200 $\mu$g of copolymer) and the body distribution of radioactivity examined after 1 h, 5 h, 24 h and 7 days. Animals were maintained in metabolic cages and urine and faeces collected throughout.

At the end of all experimental periods animals were subjected to ether anaesthetic. To assess the radioactivity remaining in the peritoneal cavity, animals were injected i.p. with saline (2 ml). After agitation of the abdomen this solution was retrieved (as far as possible) using a Pasteur pipette and assayed for radioactivity. A 50 $\mu$l blood sample was taken by puncturing the heart and then dispersed in 1 M NaOH (1 ml) and assayed for radioactivity. All the major organs were removed and washed in ice cold saline: liver, lungs, kidneys, spleen, stomach, intestines, colon and rectum. Organs were routinely homogenized to a known volume in water or, in the case of stomach and other tissues from the gastrointestinal tract, dissolved in a known volume of 1 M NaOH. All samples, including urine and faeces (dispersed to a known volume) were assayed for radioactivity. The total radioactivity recovered from each organ, urine, faeces and blood (assuming a blood volume equivalent to 5.77 ml/100 g of mouse (Dreyer & Ray, 1910) was calculated and these values summed to give the total radioactivity recovered from the body at each time. This value was also expressed as a percentage of the dose administered.

*Body distribution of* [$^3$*H*]*DNM conjugates*

Body distributions were assessed essentially as described above. Under ether anaesthetic, $DBA_2$ mice were injected intraperitoneally with [$^3$H]DNM (0.1 ml, $\sim$2.5 $\mu$g), sample 7 (0.2 ml, $\sim$260 $\mu$g conjugate), or sample 8 (0.2 ml, $\sim$260 $\mu$g conjugate). The animals were allowed to recover and maintained in metabolic cages for 1, 5 or 24 h. At the end of the experimental period, a 50 $\mu$l blood sample (dispersed in 1 M NaOH, 1.0 ml) was taken, the animal sacrificed and organs (as described above) removed. All samples were prepared as described previously and assayed for radioactivity. Homogenates (0.5 ml) and samples of urine, faeces and blood (0.5 ml) were mixed with 4.5 ml of a complete scintillant cocktail (Liquiscint) and counted for 10 min. Each sample was subsequently spiked with a [$^3$H] standard ($\sim$20,000 cpm added to each vial) and re-counted in order to assess the extent of quenching in each sample. The measured radioactivity in each sample was then corrected for the degree of quenching and the body distribution expressed in the same way as described for $^{125}$I-labelled polymers.

*Analysis of radioactivity recovered from urine and peritoneal washings using Sephadex G-25 chromatography*

Following certain body distribution experiments radioactivity recovered in the urine or peritoneal washings was subjected to Sephadex G-25 chromatography. Samples were applied to a disposable PD-10 column (Pharmacia) and eluted with 0.05 M sodium acetate (0.5 ml fractions). The columns were calibrated with blue-Dextran, $^{125}$I- and [$^3$H]DNM.

### Results

$DBA_2$ mice inoculated i.p. with $10^5$ L1210 cells died consistently after 14–17 days (Figures 2–5; Tables II–IV). Over the experimental period these animals showed a steady weight gain (Figure 2) up to $\sim$140% of the starting weight.

**Figure 2** Effect of free daunomycin on the weight, and survival, of $DBA_2$ mice inoculated i.p. with $10^5$ L1210 cells. The L1210 cells were given on day 0 and DNM administered i.p. on day 1 at doses of 0 (●), 0.25 (▲), 0.5 (■), 0.75 (○), 2 (□) or 5 mg kg$^{-1}$ (△). All animals died, and the time (days) when the last member of the group died is indicated.

**Table II** Survival of $DBA_2$ mice bearing i.p. L1210 leukaemia following i.p. treatment with DNM

| Treatment | Dose ($mg\,kg^{-1}$) | Day of death | Mean survival (±s.e.) | Long term survivors |
|---|---|---|---|---|
| None[a] | – | 15, 15, 16, 16, 17 | 15.8±0.4 | 0/5 |
| DNM | 0.25 | 14, 15, 15, 20, 22 | 17.2±1.6NS | 0/5 |
| DNM | 0.5 | 17, 21, 21, 22, 27 | 21.6±1.6* | 0/5 |
| DNM | 0.75 | 19, 19, 21, 22, 22 | 20.6±0.7* | 0/5 |
| DNM | 2.0 | 15, 16, 19, 19, 23 | 18.4±1.4NS | 0/5 |
| DNM | 5.0 | 10, 10, 10, 13, 13 | 11.2±0.7** | 0/5 |

[a]DNM was administered on day 1 after i.p. inoculation of $10^5$ L1210 cells. *$P<0.01$; **$P<0.001$.

Administration of a single, i.p. dose of free DNM (0.25–5.0 $mg\,kg^{-1}$) had a dose-dependent effect indicated by marked weight change (Figure 2). A dose of 2 $mg\,kg^{-1}$ produced slight weight loss, 5 $mg\,kg^{-1}$ caused rapid weight loss (to 76% of the starting weight), whereas lower doses of drug, 0.75, 0.5 and 0.25 $mg\,kg^{-1}$, did not prevent tumour-associated weight gain. Although certain doses did produce a significant increase in the mean survival time (Table II), the maximum increase was limited to ~135% of that recorded for the untreated control group.

Initial experiments carried out to evaluate the effectiveness of HPMA copolymer-DNM conjugates used a treatment regime comprising three i.p. doses of conjugate given on days 1, 2 and 3 after i.p. inoculation of $10^5$ L1210 cells. Survival times (Figure 3 and Table III) and the weight

**Figure 3** Effect of HPMA-daunomycin on the survival of $DBA_2$ mice inoculated i.p. with $10^5$ L1210 cells. L1210 cells were administered on day 0 followed by DNM, or HPMA copolymer-DNM given i.p. on days 1, 2 and 3. Panel (a) shows the survival of untreated mice (——) and mice treated with DNM, 2 $mg\,kg^{-1}$ (– – – –) or 5 $mg\,kg^{-1}$ (–·–·–). The survival of animals treated with conjugate 3 (d), conjugate 4 (b) and conjugate 5 (c) is also shown.

**Table III** Survival of $DBA_2$ mice bearing i.p. L1210 leukaemia following i.p. treatment with HPMA copolymer-DNM conjugates (3 doses)

| Treatment[a] | Polymer side-chain | Experiment 1 Day of death | Experiment 1 Mean[b] survival (±s.e.) | Experiment 1 Long term[b] survivors | Experiment 2 Day of death | Experiment 2 Mean survival (±s.e.) | Experiment 2 Long term survivors |
|---|---|---|---|---|---|---|---|
| None | – | 14, 15, 16, 16, 17, 17, 18, 19 | 16.5±0.6 | 2/10 | 17, 17, 17, 17, 17 | 17.0±0 | 0/5 |
| DNM | – | 10, 10, 10, 10, 10 | 10 ±0** | 0/5 | 8, 9, 10, 10, 17 | 10.8±1.6* | 0/5 |
| Sample 3 | P-Gly-Gly-DNM | 17, 17, 17, 19 | 17.5±0.5NS | 1/5 | – | – | |
| Sample 4 | P-Gly-Phe-Leu-GlyDNM | 24, 38 | 31 | 3/5 | 18, 22, 23, 25, 28 | 23.2±1.7* | 0/5 |
| Sample 5 | P<Gly-Phe-Leu-GlyDNM / Gly-Phe-Leu-GlyFucosylamine | 21, 18 | 25 | 1/3 | – | – | |
| Sample 6 | P<Gly-Phe-Leu-GlyDNM / Gly-Phe-Leu-GlyGalactosamine | – | – | | 23, 24, 30, 32, 40 | 29.8±3.1* | 0/5 |

[a]DNM (5 $mg\,kg^{-1}$), either free drug or an equivalent amount of drug bound to HPMA copolymer, was administered on days 1, 2 and 3 after i.p. inoculation of $10^5$ L1210 cells; [b]Mean survival time (±s.e.) of animals dying during the course of the experiment. The observation period was terminated at 50 days and the number of survivors is shown. *$P<0.001$; **$P<0.01$.

changes of the animals in each group (Figure 4) are shown. Free DNM (3 doses) at a daily dose of 2 or 5 mg kg$^{-1}$ was unable to prolong survival time; indeed the higher dose significantly decreased life expectancy (Figure 3). Administration of HPMA copolymer-DNM conjugates affected the survival time of L1210-bearing mice, in a manner related to the conjugate composition (Table III). The non-degradable P-Gly-Gly-DNM conjugate (sample 3) caused no significant increase in lifespan. In contrast the biodegradable conjugates containing P-Gly-Phe-Leu-Gly-DNM (samples 4, 5 and 6), produced an increase in the mean lifespan (of those animals that died during the course of the experiment) and, in certain experiments, a number of animals survived.

Weight changes observed in mice treated with either free, or conjugated, DNM were consistent with their measured survival-times (Figure 4). The animals treated with free DNM (3 doses in this case) all lost weight and died early in the experiment. Both those treated with the non-biodegradable conjugate (sample 3), and the untreated control group, gained weight rapidly, and also showed visual evidence of their rapidly growing peritoneal ascites. However, animals treated with biodegradable HPMA copolymer-DNM conjugates (with or without L-fucosylamine) showed little or no weight-change. Those animals dying within the experimental period did eventually succumb to the tumour.

Effect of the timing, and size of the dose administered is shown in Table IV. A single dose of P-Gly-Phe-Leu-Gly-DNM (sample 4) given on day 1 or day 3 was sufficient to increase the mean survival-time considerably. However, when administered on day 5 or day 8 a single dose of sample 4 produced no significant increase in mean survival time. Administration of sample 4 at different doses on day 1 or day 3 (Table IV, experiments 1 and 3) showed doses in the range 5.0–20 mg kg$^{-1}$ to be similarly effective (with a slight increase in activity as the dose administered increased).

**Figure 4** Effect of free daunomycin and HPMA copolymer-daunomycin administered i.p. on the weight of $DBA_2$ mice inoculated L1210 cells on day 0. Drug, or drug conjugate, was administered i.p. on days 1, 2 and 3; DNM 2 mg kg$^{-1}$ (○); DNM 5 mg kg$^{-1}$ (●); conjugate 3 (■); conjugate 4 (▲) or conjugate 5 (△). Untreated control animals are also shown (□) and all conjugates were given at a dose of 5 mg kg$^{-1}$ in respect of the contained DNM.

**Table IV** Survival of $DBA_2$ mice bearing i.p. L1210 leukaemia after i.p. treatment with DNM or HPMA copolymer-DNM. Effect of dose and timing of administration

| *Experiment* | *Treatment* — *Sample no.* | *Treatment* — *Polymer side-chain* | *Dose (mg kg$^{-1}$)* | *Day of admin.* | *Day of death* | *Mean survival (±s.e.)* | *Long term survivors* |
|---|---|---|---|---|---|---|---|
| *1. Administration on days 1 or 3* | | | | | | | |
| | None | – | – | – | 15, 15, 15, 15, 15, 16, 16<br>17, 20, 20 | 16.4±0.6 | 0/10 |
| | DNM | – | 5.0 | 1 | 15, 15, 16, 16, 23 | 17.0±1.5NS | 0/5 |
| | 4 | P-Gly-Phe-Leu-Gly-DNM | 2.5 | 1 | 15, 17, 19, 20, 20, 20, 20, 22 | 19.1±0.8* | 0/8 |
| | 4 | P-Gly-Phe-Leu-Gly-DNM | 5.0 | 1 | 21, 24, 24, 28, 29, 30, 39 | 27.8±2.3*** | 1/8 |
| | 4 | P-Gly-Phe-Leu-Gly-DNM | 7.5 | 1 | 21, 23, 24, 24, 26, 27, 43 | 26.8±2.8*** | 1/8 |
| | 4 | P-Gly-Phe-Leu-Gly-DNM | 5.0 | 3 | 21, 22, 25, 28, 31, 32 | 26.5±1.9*** | 0/6 |
| | 4 | P-Gly-Phe-Leu-Gly-DNM | 7.5 | 3 | 27, 28, 29, 35, 38, 38 | 32.5±2.1*** | 2/8 |
| | 5 | P<Gly-Phe-Leu-Gly-DNM<br>Gly-Phe-Leu-Gly-Fucosylamine | 7.5 | 1 | 25, 26, 26, 32, 32, 34 | 29.2±1.6*** | 0/6 |
| | 5 | P<Gly-Phe-Leu-Gly-DNM<br>Gly-Phe-Leu-Gly-Fucosylamine | 7.5 | 3 | 28, 31, 33 | 30.7±1.5*** | 4/6 |
| *2. Administration on days 1, 5 or 8* | | | | | | | |
| | None | – | – | – | 15, 15, 17, 18, 18 | 16.6±0.7 | 0/5 |
| | DNM | – | 2.0 | 1 | 19, 19, 22, 22, 23 | 21.0±0.8** | 0/5 |
| | HPMA<br>DNM | – | 20.0<br>2.0 | 1 | 12, 17, 18, 18, 23 | 17.6±1.7NS | 0/5 |
| | 4 | P-Gly-Phe-Leu-Gly-DNM | 5.0 | 1 | 19, 20, 22, 22, 25 | 21.6±1.0** | 0/5 |
| | 4 | P-Gly-Phe-Leu-Gly-DNM | 5.0 | 5 | 15, 17, 17, 18, 18 | 17.0±0.5NS | 0/5 |
| | 4 | P-Gly-Phe-Leu-Gly-DNM | 5.0 | 8 | 17, 17, 18, 18, 18 | 17.6±0.2NS | 0/5 |
| *3. Administration on day 3* | | | | | | | |
| | None | – | – | | 16, 17, 17, 17, 17 | 16.8±0.2 | 0/5 |
| | 4 | P-Gly-Phe-Leu-Gly-DNM | 5.0 | 3 | 20, 24, 25, 34 | 25.8±3.0* | 1/5 |
| | 4 | P-Gly-Phe-Leu-Gly-DNM | 7.5 | 3 | 16, 19, 19, 23, 25 | 20.4±1.6* | 0/5 |
| | 4 | P-Gly-Phe-Leu-Gly-DNM | 10.0 | 3 | 17, 22, 26 | 21.6±2.6* | 2/5 |
| | 4 | P-Gly-Phe-Leu-Gly-DNM | 15.0 | 3 | 20, 23, 24, 26, 33 | 25.2±2.2*** | 0/5 |
| | 4 | P-Gly-Phe-Leu-Gly-DNM | 20.0 | 3 | 20, 21, 23, 25, 26 | 23.0±1.1*** | 0/5 |

*$P<0.05$; **$P<0.01$; ***$P<0.001$.

In contrast the lower dose of 2.5 mg kg$^{-1}$ was totally ineffective.

Sample 5 (containing L-fucosylamine) was not obviously more effective than sample 4 when given on day 1. However, when administered on day 3, sample 5 appeared more active producing a larger number of long term survivors. Administration of polymer-bound DNM up to doses of 20 mg kg$^{-1}$ produced no visual signs of toxicity and measured animal weights in these experiments did not show any signs of sudden weight loss such as those associated with acute DNM toxicity (cf. Figures 2 & 5).

Untreated animals inoculated s.c. with L1210 died with a mean survival time of 16–20 days (Table V). Again free DNM did not improve this situation. Although, polymer-bound DNM (Sample 4) administered i.p. was not effective at 5 mg kg$^{-1}$ (a concentration shown previously to act against i.p. tumour), concentrations of 10–20 mg kg$^{-1}$ significantly increased the mean survival time and also give rise to long term survivors.

$^{125}$I-Labelled samples 4 and 5, administered i.p. showed different body distributions during the first 24 h (Figures 5 & 6). Both left the peritoneal cavity rapidly, 50% leaving within the first hour. However, the polymer containing L-fucosylamine residues (sample 5) seemed to pass more readily from the bloodstream into the kidney. At 24 h both polymers showed an almost identical body distribution, with 76% of radioactivity recovered from sample 5 in kidney or urine, and likewise 65% of sample 4 (Figure 7). Sephadex G-25 chromatography of urine collected during the 24 h following i.p. administration of $^{125}$I-labelled sample 5 showed >60% of radioactivity in urine to be in macromolecular form (Figure 8). After 7 days almost all of the administered sample 4 and 5 had been excreted.

$^{125}$I-Labelling of tyrosinamide residues in the polymer backbone is limited as it permits only tracing of the polymeric carrier. Studies were therefore carried out using [$^3$H]DNM and HPMA copolymers containing [$^3$H]DNM to monitor drug fate, both in free and conjugated form. After i.p. administration, body distributions were assessed (1, 5 and 24 h) and results obtained are shown in Table VI. Free [$^3$H]DNM showed substantial association with the intestine (49% of the radioactivity recovered after the first hour), and subsequently radioactivity appeared in the faeces. Both [$^3$H]DNM and sample 7 showed some evidence of liver association, and this was particularly noticeable in the case of D-galactosamine-containing conjugate (sample 7), 32% of the radioactivity recovered being detected in the liver after 1 h and (29%) after 5 h. Sample 8 did not accumulate to any significant extent in the liver.

It is interesting to compare the rate of excretion of free

**Table V** Survival of $DBA_2$ mice bearing s.c. L1210 leukaemia following i.p. treatment with DNM or HPMA copolymer-DNM

| *Treatment*$^a$ | *Dose* (*mg kg*$^{-1}$) | *Day of death* | *Mean survival time* (±*s.e.*) | *Long term survivors* | *Increase in mean survival time* (%) |
|---|---|---|---|---|---|
| None | – | 16, 19, 19, 23, 23, 23 | 20.5±1.1 | 0/6 | – |
| (Expt. 2) | – | 16, 16, 16, 16, 16 | 16.0±0 | 0/5 | – |
| DNM | 5 | 9, 9, 9, 10, 24 | 12.2±2.9* | 0/5 | 60 |
| Sample 4 (P-Gly-Phe-Leu-Gly-DNM) | 5 | 20, 23, 24, 24, 35 | 25.2±2.5NS | 0/5 | 123 |
| (Expt. 2) | 5 | 19, 20, 20, 22, 25 | 21.2±1.1NS | 0/5 | 133 |
| | 10 | 24, 25, 27, 37 | 28.3±3.0* | 1/5 | 138 |
| | 15 | 25, 32, 49 | 35.3±7.1* | 2/5 | 172 |
| | 20 | 22, 22 | 22 | 2/4 | 107 |

$^a$DNM and HPMA copolymer-DNM were administered on days 1, 2 and 3 after tumour inoculation; *$P<0.05$.

**Figure 5** Effect of dose on animal weight after i.p. administration of P-Gly-Phe-Leu-Gly-DNM to $DBA_2$ mice bearing i.p. L1210. L1210 cells were administered i.p. on day 0 and HPMA copolymer-DNM on day 3; no treatment (●——●); 5 (○---○); 10 (○——○); 15 (●---●) and 20 mg kg$^{-1}$ (■——■). The percent weight change is shown and also the day of death of the last surviving animal in each group.

**Figure 6** Body distribution of radioactivity after i.p. injection of $^{125}$I-labelled copolymer 4 to $DBA_2$ mice. The radioactivity recovered in each organ was expressed as a percentage of the total radioactivity recovered from the animal (mean ± s.e. of 3 animals). Radioactivity recovered from the peritoneal cavity (○---○), urine and faeces (■——■), blood (●——●), kidney (▲——▲), gastrointestinal tract (□---□) and liver (△---△) is shown.

**Figure 7** Body distribution of radioactivity after i.p. injection of $^{125}$I-labelled copolymer 5. The radioactivity recovered in each organ was expressed as a percentage of the total radioactivity recovered from the animal (mean ± s.e. of 3 animals). Radioactivity recovered in the various organs is indicated according to the key shown in the legend to **Figure 6**.

and conjugated [$^{3}$H]DNM. For free [$^{3}$H]DNM, 46% of the radioactivity recovered after 24 h was in the urine and faeces. This compares with 72% of sample 7 and 73% of sample 8. If these results are related to the dose administered, only 10.2% of free [$^{3}$H]DNM is excreted within 24 h compared with 29% and 35% of samples 7 and 8 respectively. Sephadex G-25 chromatography of peritoneal samples 1 h after administration showed two peaks corresponding to either low or high molecular weight material and, representing [$^{3}$H]DNM and conjugate respectively (Table VII).

After 24 h free [$^{3}$H]DNM was detectable in the urine samples of animals given either free drug or sample 7 (Figure 9). Correspondingly little free [$^{3}$H]DNM was detected in the urine of animals given sample 8.

**Figure 8** Sephadex G-25 elution of mouse urine collected 24 h after i.p. administration of $^{125}$I-labelled copolymer 5 procedure is given in **Materials and methods**. Elution of dextran blue ($V_0$) and $^{125}$I- is shown.

**Figure 9** Sephadex G-25 elution of mouse urine collected 24 h after i.p. administration of (a) [$^{3}$H]DNM; (b) sample 7; (c) sample 8. Procedure is given in **Materials and methods**. Elution of dextran blue ($V_0$) and [$^{3}$H]DNM is also shown.

**Table VI** Body distribution of [$^3$H]DNM and HPMA-[$^3$H]DNM (samples 7 and 8) after i.p. administration

| Organ | *Time*[a] | | | | | | | | |
|---|---|---|---|---|---|---|---|---|---|
| | *1 h* | | | *5 h* | | | *24 h* | | |
| | [$^3$*H*]*DNM* | *Sample 7* | *Sample 8* | [$^3$*H*]*DNM* | *Sample 7* | *Sample 8* | [$^3$*H*]*DNM* | *Sample 7* | *Sample 8* |
| Liver | 15.2 | 31.6 | 2.9 | 13.7 | 28.6 | 2.6 | 8.0 | 7.6 | 4.6 |
| Lung | 0.6 | 0.9 | 0.6 | 1.3 | 1.8 | 0.8 | 0.5 | 0.5 | 0.6 |
| Kidney | 2.6 | 6.5 | 1.9 | 8.3 | 8.5 | 2.3 | 14.2 | 3.8 | 1.0 |
| Spleen | 0.7 | 0.4 | 0.4 | 2.5 | 1.6 | 0.3 | 1.3 | 0.8 | 0.6 |
| Heart | 0.5 | 0.5 | 0.4 | 0.8 | 0.4 | 0.5 | 0.5 | 0.5 | 0.5 |
| Stomach | 4.8 | 1.9 | 1.2 | 2.8 | 5.6 | 1.2 | 3.3 | 0.9 | 0.5 |
| Intestine | 48.6 | 14.4 | 6.9 | 43.1 | 23.0 | 6.6 | 18.7 | 8.5 | 8.9 |
| Peritoneal washings | 22.1 | 28.1 | 63.2 | 5.6 | 9.9 | 3.7 | 1.9 | 0.7 | 1.3 |
| Urine | –[b] | – | – | 11.4 | 1.8 | 48.3 | 11.4 | 40.9 | 60.0 |
| Faeces | – | – | – | 6.2 | 16.9 | 14.8 | 34.7 | 30.6 | 12.7 |
| Blood | 5.0 | 15.9 | 22.5 | 4.3 | 1.8 | 18.9 | 6.0 | 5.2 | 9.3 |
| Radioactivity recovered as a percentage of dose administered | 48 | 52 | 50 | 26 | 29 | 43 | 22 | 41 | 48 |

[a]The radioactivity recovered in each organ is expressed as a percentage of the total radioactivity recovered from the animal. Recovery in respect of dose administered is also shown; [b]Values not determined.

**Table VII** Sephadex G-25 chromatography of urine and peritoneal washings after administration of [$^3$H]DNM, sample 7 or sample 8

| *Sample administered* | *Length of experiment* (*h*) | *Percentage of radioactivity* ([$^3$H]) *recovered from the column*[a] | |
|---|---|---|---|
| | | *Peak 1* | *Peak 2* |
| *Peritoneal washings* | | | |
| [$^3$H]DNM | 1 | 28.3 | 63.9 |
| | 5 | 1.8 | 98.2 |
| Sample 7 | 1 | 81.6 | 18.4 |
| | 5 | –[b] | – |
| Sample 8 | 1 | 98.4 | 156 |
| *Urine* | 5 | – | – |
| [$^3$H]DNM | 5 | 6.4 | 93.6 |
| | 24 | 16.1 | 83.9 |
| Sample 7 | 5 | – | – |
| | 24 | – | – |
| Sample 8 | 5 | 98.3 | ND[c] |
| | 24 | 70.2 | 28.4 |

[a]Radioactivity eluting in peak 1 (Void volume) and a second peak of lower molecular weight material expressed as a percentage of the total radioactivity eluting from the column, see **Figure 9**; [b]These values were not determined; [c]Not detectable.

### Discussion

Modification of anthracycline antibiotic disposition with the aid of drug-carriers can produce an increase in a drug's therapeutic index, and in certain cases overcome cell resistance (Yanovich *et al.*, 1984). This has been demonstrated with anthracyclines using a number of different carriers, including liposomes (Fichtler *et al.*, 1984), low density lipoprotein (Yanovich *et al.*, 1984), and DNA or protein carriers (Trouet & Jolles, 1984). In this study we have shown that a soluble synthetic polymer may also have potential as a carrier of anticancer agents. Administration of DNM bound to HPMA copolymer, via a lysosomally degradable tetrapeptide (Gly-Phe-Leu-Gly), increased the mean survival-time (relative to untreated controls) of $DBA_2$ mice bearing L1210 leukaemia and also produced a number of animals surviving long-term (Figure 3 and Tables III & IV). These data are in accord with previous studies describing successful treatment of i.p. tumours using i.p. administration of degradable drug conjugates: non-covalently linked DNA (Deprez-de Campeneere & Trouet, 1980), poly-L-aspartic acid and poly-L-lysine (Zunino *et al.*, 1984), poly-L-aspartic acid (Pratesi *et al.*, 1985) and succinylated fetuin or albumin (Trouet *et al.*, 1982).

There are at least two mechanisms of action of macromolecular drug conjugates (Duncan, 1987). Conjugation of a drug alters its pharmacokinetics and this simple procedure can exclude drug from the principle sites of toxicity thus improving the therapeutic index considerably. Secondly, conjugation limits capture of drug to the cellular uptake process of pinocytosis, affording potential to target to particular cells using cell-specific surface receptors or antigens to promote capture by receptor-mediated pinocytosis. Internalization of a drug-conjugate in this way ultimately results in its exposure to lysosomal enzymes and therefore lysosomally degradable conjugates can liberate drug intracellularly following uptake. As yet there are no convincing examples of significant, tumour-specific, drug targeting, even with the use of tumour-specific monoclonal antibodies as drug-carriers. Most drug conjugates currently proposed probably exert their effect by modulation of the body distribution, and rate of excretion, of the attached agent.

Throughout this investigation sample 3, P-Gly-Gly-DNM, shown previously (Kopeček & Duncan, 1987*a*) to be non-biodegradable, was found to be completely inactive. In contrast samples 4, 5 and 6, containing biodegradable drug-carrier linkages, all displayed marked antitumour activity. The stability of sample 8 was confirmed as almost all the radioactivity recovered in the urine after i.p. administration was macromolecular, i.e., polymer-bound[$^3$H]DNM. In contrast Sephadex G-25 chromatography of urine following administration of sample 7 showed a large peak of low molecular weight material coeluting with [$^3$H]DNM. (Figure 9.) These observations suggest that drug release from HPMA copolymer conjugates by enzymic hydrolysis is a prerequisite for pharmacological activity. Inability of rat plasma and serum to hydrolyse such HPMA copolymers (Rejmanová *et al.*, 1985); and the known ability of lysosomal enzymes to cleave these substrates (Rejmanová *et al.*, 1983; Duncan *et al.*, 1983) suggests that drug release occurs intracellularly. Trouet *et al.* (1982) showed that albumin-DNM, and albumin-Ala-Leu-DNM, were much less active against L1210 *in vivo* than albumin-Ala-Leu-Ala-Leu-DNM, an observation that was attributed to resistance of the former to hydrolysis by lysosomal enzymes. However, ability of anthracycline antibiotics to induce toxicity via interaction with the cell surface should not be overlooked. Tokes *et al.* (1982) and Tritton & Lee (1982) attached adriamycin to polyglutaraldehyde microspheres, and agarose respectively, and were able to show that non-penetrating drug formulations can be toxic to tumour cells *in vitro*, including L1210 leukaemia. Previous *in vitro* studies with HPMA copolymer conjugates (Duncan *et al.*, 1987) showed differential toxicity of polymer conjugates containing stable or biodegradable DNM-polymer linkages to L1210 cells. The non-degradable conjugates were slightly toxic, but much less so than biodegradable ones, a factor attributed to cell-surface activity. In these *in vivo* studies lack of activity of non-degradable sample 3 implies that the observed antitumour activity of biodegradable drug-conjugate is not simply caused by cell surface interaction.

Inoculation of tumour intraperitoneally, followed by i.p. treatment may apparently confine the tumour and treatment to one body compartment, thus biasing the investigation in favour of tumour targeting, or tumour killing. However, polymer-DNM administered i.p. was also shown to be active against s.c. implanted tumour (Table V) and the ability of the polymeric conjugate to act at a site remote from the point of administration is consistent with the short half-life of radiolabelled HPMA copolymer-DNM conjugates in the peritoneal cavity (Figures 6 & 7), demonstrated previously (Seymour *et al.*, 1987*a*), to be independent of polymer molecular weight over the range 10,000–800,000.

HPMA copolymer-drug conjugates, either $^{125}$I-labelled or containing [$^{3}$H]DNM were eliminated from the body more readily than [$^{3}$H]DNM (Table VI; Figures 6 & 7). Taking into account total recovery of radioactivity in respect of administered dose, the rate of excretion of [$^{3}$H]DNM was more than three times greater when attached to polymer. Attachment of DNM to polymer prevents random intracellular access and therefore rapid binding of drug to intracellular constituents such as DNA, and as the molecular weight of the polymer conjugate is sufficiently small to pass across the kidney glomerulus any conjugate resident in, or passing into, the circulation was quickly removed. This may in part explain the decreased toxicity of polymer-bound drug. (Doses of polymer-bound drug up 20 mg kg being administered without any obvious ill effect.) The fact that polymer-bound anthracyclines show less toxicity to human lymphocytes and mouse spleen cells *in vitro* (Říhová, unpublished) and are probably less cardiotoxic will also contribute to the overall reduction in toxicity of conjugated drug. It is noteworthy that the more rapid excretion of a dose of polymer-drug is still accompanied by a measurable therapeutic response.

The HPMA copolymer bearing [$^{3}$H]DNM and galactosamine (sample 7) showed greater association with the liver (Table VI) than polymer without the sugar, almost certainly due to receptor-mediated pinocytic uptake by hepatocytes (Ashwell & Harford, 1982; Duncan *et al.*, 1986). DNM subsequently released from this intracellular depot was effective against L1210 inoculated i.p. (Table III). This is perhaps surprising as the liver is known to be the primary site of DNM metabolism (Craddock *et al.*, 1973) and it could be predicted that any DNM released from hepatocyte lysosomes might be inactivated. This is clearly not the case and these observations suggest that targeted HPMA copolymers bearing anthracyclines have potential, following i.v. bolus administration, as a controlled release depot in the liver. Anthracycline released could be used to treat primary (known also to retain the galactose-recognising receptor, Schwartz *et al.*, 1982) and secondary liver cancer. Two lethal diseases. HPMA copolymer-DNM conjugates administered i.v. to mice were not non-specifically hepatotoxic as measured by mouse weight and their plasma transaminase/alkaline phosphatase levels (McCormick *et al.*, 1987).

Substitution of conjugates with the other carbohydrate residue fucosylamine, did not increase the mean survival of animals in comparison with treatment using non-carbohydrate conjugate (Table IV), the only exception being after administration of single doses (7.5 mg kg$^{-1}$) on day 3. The explanation of this observation is not clear, but could indicate greater expression of the fucose receptor during the rapid growth phase of the developing ascites.

Recent experiments have shown that HPMA copolymer-adriamycin conjugates are more effective than the described DNM conjugates in prolonging life of $DBA_2$ mice inoculated with L1210 cells (Kopeček & Duncan, 1987*b*). After i.v. administration they do not manifest toxicity until a dose of 75 mg kg$^{-1}$. As this anthracycline is one of the most important clinical agents, such HPMA copolymer conjugates appears to have real therapeutic potential. Unlike many of the other drug-carriers evaluated in this context this polymer and its conjugates are not immunogenic (Říhová *et al.*, 1984) and so can be administered repeatedly. Use of techniques in polymer chemistry to synthesize drug conjugates prevents either denaturation of the carrier or inactivation of drug.

We thank the Cancer Research Campaign for funding this work and The Royal Society and British Council for supporting the international collaboration.

### References

ASHWELL, G. & HARFORD, J. (1982). Carbohydrate-specific receptors of the liver. *Ann. Rev. Biochem.*, **51**, 531.

CHENG, P.W. & BOAT, T.F. (1978). An improved method for the determination of galactosaminitol, glucosamine and galactosamine on an amino acid analyzer. *Anal. Biochem.*, **85**, 276.

CHU, B. & HOWELL, S.B. (1981). Pharmacological and therapeutic properties of carrier bound methotrexate against tumor confined to a third space body compartment. *J. Pharmacol. Exp. Ther.*, **219**, 389.

CHU, B., FAN, C.C. & HOWELL, S.B. (1981). Activity of free and carrier-bound methotrexate against transport-deficient and high dihydrofolate dehydrogenase-containing methotrexate-resistant L1210 cells. *J. Natl Cancer Inst.*, **66**, 121.

CRADOCK, J.C., EGORIN, M.J. & BACHUR, N.R. (1973). Daunorubicin biliary excretion and metabolism in the rat. *Arch. Int. Pharmacodyn. Ther.*, **202**, 48.

DEPREZ-DE CAMPENEERE, D. & TROUET, A. (1980). DNA-anthracycline complexes. I. Toxicity in mice and chemotherapeutic activity against L1210 leukaemia of daunorubicin-DNA and adriamycin-DNA. *Eur. J. Cancer*, **16**, 981.

DREYER, G. & RAY, W. (1910). The blood volume of mammals as determined by experiments upon rabbits, guinea pigs and mice: And its relationship to the body weight and surface area expressed as a formula. *Philos. Trans. R. Soc. London Ser.*, **201**, 133.

DUNCAN, R., CABLE, H., LLOYD, J.B., REJMANOVÁ, P. & KOPEČEK, J. (1983). Polymers containing enzymatically degradable bonds, 7. Design of oligopeptide side-chains in poly N-(2-hydroxypropyl)methacrylamide copolymers to promote efficient degradation by lysosomal enzymes. *Makromol. Chem.*, **184**, 1997.

DUNCAN, R., CABLE, H.C., REJMANOVÁ, P., KOPEČEK, J. & LLOYD, J.B. (1984). Tyrosinamide residues enhance pinocytic capture of N-(2-hydroxypropyl)methacrylamide copolymers. *Biochim. Biophys. Acta*, **799**, 1.

DUNCAN, R. & KOPEČEK, J. (1984). Soluble synthetic polymers as potential drug carriers. *Adv. Polymer Sci.*, **57**, 51.

DUNCAN, R., SEYMOUR, L.C.W., SCARLETT, L., LLOYD, J.B., REJMANOVÁ, P. & KOPEČEK, J. (1986). Fate of N-(2-hydroxypropyl)methacrylamide copolymers with pendent glactosamine residues after i.v. administration to rats. *Biochim, Biophys., Acta*, **880**, 62.

DUNCAN, R. (1987). Selective endocytosis of macromolecular drug carriers: In *Sustained and Controlled Release Drug Delivery Systems*, Lee, V.H.L. & Robinson, J.R. (eds) p. 581. Marcel Dekker: New York.

DUNCAN, R., KOPEČKOVÁ-REJMANOVÁ, P., STROHALM, J., HUME, I., LLOYD, J.B. & KOPEČEK, J. (1987). Anticancer agents coupled to N-(2-hydroxypropyl)methacrylamide copolymers 1. Evaluation of daunomycin and puromycin conjugates *in vitro*. *Br. J. Cancer*, **55**, 165.

FICHTLER, J., ARNDT, D., ELBE, B. & RESZKA, R. (1984). Cardiotoxicity of free and liposomally encapsulated rubomycin in mice. *Oncology*, **41**, 363.

GARNETT, M.C., EMBLETON, M.J., JACOBS, E. & BALDWIN, R.W. (1985). Studies on the mechanism of action of an antibody-targeted drug carrier conjugate. *Anti-Cancer Drug Design*, **1**, 3.

GERAN, R.I., GREENBERG, N.H., MACDONALD, M.M., SCHUMACHER, A.M. & ABBOTT, B.J. (1972). Protocols for screening chemical agents and natural products against animal tumours and other biological systems. *Cancer Chemotherap. Rep.*, **3**, 1.

KOIJMA, T., HASHIDA, M., MURANISHI, S. & SEZAKI, H. (1980). Mitomycin C-dextran conjugate: A novel high molecular weight prodrug of mitomycin C. *J. Pharm. Pharmacol.*, **32**, 30.

KOPEČEK, J. (1977). Reactive copolymers of N-(2-hydroxypropyl)methacrylamide with N-methacryloylated derivatives of L-leucine and L-phenylalanine. I. Preparation characterization and reaction with diamines. *Makromol. Chem.*, **178**, 2169.

KOPEČEK, J. & REJMANOVÁ, P. (1979). Reactive copolymers of N-(2-hydroxypropyl)methacrylamide with N-methacryloylated derivatives of L-leucine and L-phenylalanine. II. Reaction with the polymeric amine and stability of crosslinks towards chymotrypsin *in vitro*. *J. Polym. Sci. Symp.*, **66**, 15.

KOPEČEK, J., REJMANOVÁ, P., DUNCAN, R. & LLOYD, J.B. (1985*a*). Release of drug model from N-(2-hydroxypropyl)methacrylamide copolymers. *Ann. N.Y. Acad. Sci.*, **446**, 93.

KOPEČEK, J., REJMANOVÁ, P., STROHALM, J. & 5 others (1985*b*). Synthetic polymeric drugs. *British Patent App.*, 8 500 209 (4.1.85).

KOPEČEK, J. & DUNCAN, R. (1987*a*). Poly N-(2-hydroxypropyl)methacrylamide macromolecules as drug carrier systems. In *Polymers in Controlled Drug Delivery*, Illum, L. & Davis, S.S. (eds). John Wright: Bristol, U.K.

KOPEČEK, J. & DUNCAN, R. (1987*b*). Targetable polymeric prodrugs. *J. Controlled Rel.*, **6**, 315.

MCCORMICK, L.A., DUNCAN, R. & KOPEČEK, J. (1987). Biocompatibility of soluble synthetic polymers developed as drug-carriers, *2nd International Conference on Biointeractions*, p. 32. Butterworths: U.K. (*Abstract*).

MONSIGNY, M., ROCHE, A.-C. & MIDOUX, P. (1984). Uptake of neoglycoproteins via membrane lectin(s) of L1210 cells evidenced by quantitative flow cytofluorometry and drug targeting. *Biol. Cell*, **51**, 187.

PLUMMER, T.H., JR. (1976). A simplified method for determination of amino sugars in glycoproteins. *Anal. Biochem.*, **73**, 532.

PRATESI, G., SAVI, G., PEZZONI, G., BELLINI, O., TINELLI, S. & ZUNINO, F. (1985). Poly-L-aspartic acid as a carrier for doxorubicin: A comparative *in vivo* study of free and polymer-bound drug. *Br. J. Cancer*, **52**, 841.

REJMANOVÁ, P., KOPEČEK, J., POHL, J., BAUDYŠ, M. & KOSTKA, V. (1983). Polymers containing enzymatically degradable bonds, 8. Degradation of oligopeptide sequences in N-(2-hydroxypropyl)methacrylamide copolymers by bovine spleen cathepsin B. *Makromol. Chem.*, **184**, 2009.

REJMANOVÁ, P., KOPEČEK, J., DUNCAN, R. & LLOYD, J.B. (1985). Stability in rat plasma and serum of lysosomally degradable oligopeptide sequences in N-(2-hydroxypropyl)methacrylamide copolymers. *Biomaterials*, **6**, 45.

ŘÍHOVÁ, B., KOPEČEK, J., ULBRICH, K., POSPÍŠIL, J. & MANCAL, P. (1984). Effect of the chemical structure of N-(2-hydroxypropyl)methacrylamide copolymers on their ability to induce antibody formation in inbred strains of mice. *Biomaterials*, **5**, 143.

ŘÍHOVÁ, B., KOPEČEK, J. (1985). Biological properties of targetable poly[N-(2-hydroxypropyl)methacrylamide]-antibody conjugates. *J. Controlled Release*, **2**, 289.

ŘÍHOVÁ, B., KOPEČEK, J., KOPEČKOVÁ-REJMANOVÁ, P., STROHALM, J., PLOCOVÁ, D. & SEMORADOVA, H. (1986). Bioaffinity therapy with antibodies and drugs bound to soluble synthetic polymers. *J. Chromatography Biomed. Appl.*, **376**, 221.

RYSER, H.J.-P. & SHEN, W.-C. (1980). Conjugation of methotrexate to poly(L-lysine) as a potential way to overcome drug resistance. *Cancer*, **45**, 1207.

SCHWARTZ, AL.L., FRIDOVICH, S.E. & LODISH, H.F. (1982). Kinetics of internalization and recycling of the asialoglycoprotein receptor in a human hepatoma cell line. *J. Biol. Chem.*, **257**, 4230.

SEYMOUR, L.W., DUNCAN, R., STROHALM, R. & KOPEČEK, J. (1987*a*). Effect of molecular weight of N-(2-hydroxypropyl)-methacrylamide copolymers on body distribution and rate of excretion after s.c., i.p. and i.v. administration to rats. *J. Biomed. Mater. Res.*, **21**, 1341.

TAKAKURA, Y., MATSUMOTO, S., HASHIDA, M. & SEZAKI, H. (1984). Enhanced lymphatic delivery of mitomycin C conjugated with dextran. *Cancer Res.*, **44**, 2505.

TOKES, Z.A., ROGERS, K.E. & REMBAUM, A. (1982). Synthesis of adriamycin-coupled polyglutaraldehyde microspheres and evaluation of their cytostatic activity. *Proc. Natl Acad. Sci.*, **79**, 2026.

TRITON, T.R. & YEE, G. (1982). The anticancer agent adriamycin can be actively cytotoxic without entering cells. *Science*, **217**, 248.

TROUET, A., MASQUELIER, M., BAURAIN, R. & DEPREZ-DE CAMPENEERE. (1982). A covalent linkage between daunorubicin and proteins that is stable in serum and reversible by lysosomal hydrolases, as required for a lysosomtropic drug carrier conjugate: *In vitro* and *in vivo* studies. *Proc. Natl Acad. Sci.*, **79**, 626.

TROUET, A. & JOLLES, G. (1984). Targeting of daunomycin by association with DNA or proteins: A review. *Sem. Oncol.*, **II**, 64.

ULBRICH, K., KONAK, C., TUZAR, Z. & KOPEČEK, J. (1987). Solution properties of drug carriers based on poly[N-(2-hydroxypropyl)methacrylamide] containing biodegradable bonds. *Makromol. Chem.*, (in press).

ULBRICH, K., ZACHARIEVA, E.I., KOPEČEK, J., HUME, I.C. & DUNCAN, R. (1987). Polymer-bound derivatives of sarcolysin and their anti-tumour activity against mouse and human leukaemia *in vitro*. *Makromol. Chem.*, (in press).

YANOVICH, S., PRESTON, L. & SHAW, J.M. (1984). Characteristics of uptake and cardiotoxicity of a low-density lipoprotein-daunomycin complex in P388 leukemic cells. *Cancer Res.*, **44**, 3377.

ZUNINO, F., SAVI, G., GIULIANI, F. & 4 others (1984). Comparison of antitumour effects of daunorubicin covalently linked to poly-L-amino acid carriers. *Eur. J. Cancer Clin. Oncol.*, **20**, 421.